# Current News

**Published:** 1894-02

**Authors:** 


### Current News.

### RECENT PATENTS.

A LIST of recent patents reported specially for the INTERNATIONAL DENTAL JOURNAL.

508,564.—Dental Chair. Theodore N. Clark, Toronto, Canada, assignor to Frank E. Case, Canton, Ohio. Filed September 29, 1892.

508,677.—Dental Impression-Tray. Henry A. Burlingame, Pawtucket, R. I. Filed August 11, 1893.

508,692.—Rheostat for controlling Electrically-Operated Dental Apparatus. Jonathan P. B. Fiske, Lynn, assignor to the General Electric Company, Boston, Mass. Filed July 10, 1893.

509,176.—Dental Heater. Theodore C. Lewis, Buffalo, N. Y., assignor to the Buffalo Dental Manufacturing Company, same place. Filed August 14, 1893.

### WOMAN'S DENTAL ASSOCIATION OF THE UNITED STATES.

THE regular monthly meeting of the Woman's Dental Association was held November 4, 1893, at 1602 Arch Street, Philadelphia, President Mary H. Stilwell in the chair.

Dr. Mattie T. Haley read a paper entitled "How may we be Successful with Children?"

The next meeting will be held at 1300 Arch Street, December 2, 1893, at 7.30 P.M.

The regular monthly meeting of the Womans' Dental Association was held December 2, 1893, at 1300 Arch Street, Philadelphia, the President, Dr. Mary H. Stilwell, in the chair.

Dr. Edward C. Kirk read a paper entitled "Gum-Lancing in Difficult Primary Dentition."

The next meeting will be held at 1308 Walnut Street, Philadelphia, January 13, 1894, at 7.30 P.M.

The regular monthly meeting of the Woman's Dental Association was held January 13, 1894, at 1308 Walnut Street, Philadelphia, Dr. Elizabeth A. Davis, Chairman of Executive Committee, in the chair.

Dr. Martha C. Corkhill read a paper; subject, "Advisability of explaining Existing Conditions to Patients."

The next meeting will be held at 1308 Walnut Street, Philadelphia, February 3, 1894.

ELIZA YERKES,
*Recording Secretary.*

4004 CHESTNUT STREET, PHILADELPHIA.

---

### DENTAL COMMISSIONERS OF CONNECTICUT.

THE following are the commissioners under the new law passed in that State: Dr. Civilion Fones, President; Dr. George L. Parmele, Recorder; Dr. William J. Rider, Dr. Charles P. Graham, Dr. Richard W. Browne.

---

### ST. LOUIS DENTAL SOCIETY.

THE following officers for the year 1894 were elected January 2, 1894:

President, Dr. J. B. Newby; Vice-President, Dr. J. B. Vernon; Recording Secretary, Dr. F. F. Fletcher; Corresponding Secretary, Dr. John G. Harper; Treasurer, Dr. A. J. Prossor.

*Committee on Ethics.*—Dr. William N. Morrison, Dr. P. F. Helmuth, Dr. O. H. Manhard.

*Committee on Publication.*—Dr. L. A. Young, Dr. P. H. Eisloeffel, Dr. C. L. Pepperling.

JOHN G. HARPER,
*Corresponding Secretary.*

